# Opportunities to improve vaccination coverage in a country with a fledgling health system: Findings from an assessment of missed opportunities for vaccination among health center attendees—Timor Leste, 2016

**DOI:** 10.1016/j.vaccine.2019.06.041

**Published:** 2019-06-22

**Authors:** Anyie J. Li, Thelge Sudath Rohana Peiris, Colin Sanderson, Laura Nic Lochlainn, Manuel Mausiry, Rosye Bela Joana Benevides Moniz da Silva, Ikechukwu Udo Ogbuanu

**Affiliations:** a ASPPH/CDC Allan Rosenfield Global Health Fellowship and PHI/CDC Global Health Fellowship, Atlanta, GA, USA; b Centers for Disease Control and Prevention, Global Immunization Division, Atlanta, GA, USA; c World Health Organization, Country Office, Dili, Timor-Leste; d London School of Hygiene and Tropical Medicine, London, United Kingdom; e World Health Organization, Headquarters, Geneva, Switzerland; f Ministry of Health, Dili, Timor-Leste

**Keywords:** Missed opportunities for vaccination, Immunization, Vaccination coverage, Coverage and equity, Healthcare utilization, Timor Leste

## Abstract

**Introduction:**

Since its independence in 2002, Timor Leste has made significant strides in improving childhood vaccination coverage. However, coverage is still below national targets, and children continue to have missed opportunities for vaccination (MOV), when eligible children have contact with the health system but are not vaccinated. Timor Leste implemented the updated World Health Organization methodology for assessing MOV in 2016.

**Methods:**

The MOV data collection included quantitative (caregiver exit interviews and health worker knowledge, attitudes, practices surveys (KAP)) and qualitative arms (focus group discussions (FGDs) with caregivers and health workers and in-depth interviews (IDIs) with health administrators). During a four-day period, health workers and caregivers with children <24 months of age attending the selected eight facilities in Dili Municipality were invited to participate. The researchers calculated the proportion of MOV and timeliness of vaccine doses among children with documented vaccination histories (i.e., from a home-based record or facility register) and thematically analyzed the qualitative data.

**Results:**

Researchers conducted 365 caregiver exit interviews, 169 health worker KAP surveys, 4 FGDs with caregivers, 2 FGDs with health workers, and 2 IDIs with health administrators. Among eligible children with documented vaccination histories (n = 199), 41% missed an opportunity for vaccination. One-third of health workers (33%) believed their knowledge of immunization practices to be insufficient. Qualitative results showed vaccines were not available at all selected health facilities, and some facilities reported problems with their cold chain equipment.

**Conclusion:**

This study demonstrates that many children in Timor Leste miss opportunities for vaccination during health service encounters. Potential interventions to reduce MOV include training of health workers, improving availability of vaccines at more health facilities, and replacing unusable cold chain equipment. Timor Leste should continue to scale up successful MOV interventions beyond Dili Municipality to improve vaccination coverage nationally and strengthen the health system overall.

## 1. Introduction

Timor Leste is a small country on the eastern half of the island of Timor and is one of the world’s newest nations, having gained independence in 2002. It has a population of almost 1.3 million, of which 60% are younger than 25 years [1]. Dili municipality, which is the national capital, is one of 13 municipalities in Timor Leste. Dili is the smallest municipality in Timor Leste in terms of geographic area, but has a population of 281,000, comprising more than 20% of the total population of Timor Leste [1]. Tetun and Portuguese are the official languages of Timor Leste; about 32 indigenous languages are also spoken [1].

A civil war between 1975 and 1999 caused extensive damage to Timor Leste’s health infrastructure with more than 30% of health facilities completely destroyed [2–4]. By the end of the civil war, nearly all medical equipment had been damaged beyond use and only about 25 doctors remained in the country [2]. Immediate efforts were made in the early 2000s to rebuild the health infrastructure. Within two years of the end of the conflict, the number of health workers in the country increased to 800, and the country now has more than 1000 doctors, one national hospital (Hospital Nacional Guido Valadares [HNGV]), 5 referral hospitals, and more than 70 community health centers (CHCs) and 300 health posts [5–8].

Since gaining its independence in 2002, the government has prioritized childhood immunization [1]. Coverage of the third dose of diphtheria-tetanus-pertussis vaccine (DTP) increased from 63% in 2006 to 92% in 2017, based on official estimates and from 63% to 76% during that same time period according to World Health Organization (WHO) and United Nations Children’s Fund (UNICEF) estimates of national immunization coverage (WUENIC) [9]. Gavi, the Vaccine Alliance (Gavi) began supporting Timor Leste in 2012, following a request for financial assistance in 2011 for introduction of DTP-hepatitis B-*Haemophilus influenzae* type b vaccine (pentavalent) and in 2013 for Health Systems Strengthening (HSS) support. In 2015, Timor Leste became the final country in the Southeast Asia Region to establish a National Immunization Technical Advisory Group [10]. To further improve its immunization program, Timor Leste began a two-year “twinning” program with Sri Lanka in 2017 to learn from the Sri Lanka immunization program, which has one of the best immunization coverage rates in Southeast Asia [11,12].

Despite big improvements in immunization coverage over the past several years, children are still being missed for vaccinations during health center visits. In a secondary data analysis performed in 2016, about half (47%) of children in Timor Leste were not fully immunized, with a third of all children (30–40%) having a missed opportunity for vaccination (MOV) [13]. A MOV includes any contact with health services by a child (or adult) who is eligible for vaccination (unvaccinated, partially vaccinated/not-up-to-date, and free of contraindications) where they do not receive all the vaccine doses for which he or she is eligible [14–16]. In the first systematic literature review of MOV, the global median MOV prevalence was 32% among both children and women of childbearing age who visited a health center and 67% among the subpopulation of women and children eligible for vaccination at the time of visit [15]. In an updated systematic review published in 2014, the global median MOV prevalence remained at 32% among children and 47% among women of childbearing age who visited a health center [17].

In May 2016, Timor Leste was selected as the first country in the Southeast Asia Region to pilot the WHO methodology for assessment of MOV [18]. The main objective was to identify potential areas of improvement to reduce MOV and further improve coverage and equity by identifying and characterizing the extent of MOV among children <24 months of age attending health services.

## 2. Methods

### 2.1. Study design

The assessment of MOV in Timor Leste utilized a cross-sectional study design employing both qualitative and quantitative methods based on the WHO Planning Guide to Reduce Missed Opportunities for Vaccination (MOV) and *Methodology for the Assessment of Missed Opportunities for Vaccination* [16,18]. Past MOV assessments and the MOV guides describe the detailed process of the MOV assess ment [16,18–21]. The methodology, as implemented in Timor Leste, is described below.

Quantitative data collection included health center exit interviews (with caregivers) and knowledge, attitudes, and practices (KAP) surveys (with health workers). Qualitative data collection involved focus group discussions (FGDs) with caregivers, FGDs with health workers and in-depth interviews (IDIs) with health center administrators. The assessment concluded with a brainstorming session of interventions to reduce MOV and a high-level debrief and endorsement of an MOV intervention work plan.

### 2.2. Data collection tools

The generic WHO MOV caregiver exit questionnaire, health worker KAP survey, and FGD and IDI guides were adapted for use in Timor Leste and translated into the local language (Tetun)[18]. Caregiver exit questionnaires collected information on the demographics of the child and caregiver, the reported reason for the visit, the child’s vaccination history, and the caregiver’s knowledge of routine immunization. The health worker KAP surveys included participant demographics and health worker knowledge of vaccination and attitudes toward vaccination, with an additional section on vaccination practices targeted specifically at health workers who routinely administer vaccines. The caregiver exit questionnaires and KAP surveys included both mandatory and non-mandatory questions. FGD and IDI guides focused on understanding why opportunities for vaccination were missed and what could be done to address any problem areas.

### 2.3. Sampling

Dili Municipality was chosen as the MOV assessment area because it comprises more than 20% of the country’s total population and because of operational and logistical difficulties in reaching other municipalities [1]. As suggested in the MOV methodology, efforts were made to include both urban and rural settings and public and private facilities. The HNGV, five CHCs (Bairo Formosa, Becora, Comoro, Metinaro, Veira Cruz), and two private or nongovernmental organization (NGO) clinics (Bairo Pite and Hospital Maternidade Fatumenta) were selected for data collection. Initially, data collection was planned in all six of the CHCs in Dili Municipality, but the local MOV strategy team deemed the sixth CHC, located on the small island of Atauro, inaccessible due to transportation constraints. As a result, only one health facility included in the sample (CHC Metinaro) is considered rural, while the rest are considered urban facilities. In Timor Leste, private or NGO facilities also provide vaccination free of charge; however, they account for less than 1% of all vaccination in the country. Because private clinics are unique to Dili Municipality (as they are not found elsewhere in the country), and account for a high proportion of patient flow in Dili, they were also included in the sampling. Private or NGO facilities were purposely selected based on clinic size.

In Timor Leste, all 70 CHCs and all five referral hospitals have access to electricity and cold chain equipment and provide daily vaccination services [8]. Due to a policy requirement, the HNGV only provides vaccines to newborns. Each CHC serves 8–13 *sucos* (villages). Some sucos have health posts while others do not. There are over 300 health posts in the country [8]. A proportion of these health posts also provide daily vaccination services. For the sucos without health posts, Integrated Community Health Services (SISCa) are conducted monthly by the associated CHC. All the selected CHCs, health posts, private, and NGO health facilities for the MOV assessment offer daily vaccination services.

### 2.4. Data collection

Prior to data collection, 11 quantitative data collectors underwent three days of training in Dili on the MOV methodology and use of tablets for electronic data collection. All quantitative data were collected on tablets using survey software (Zegeba AS [Alesund, Norway]). Two qualitative data collectors also participated in the training, with a separate half-day training to familiarize them with the qualitative data collection methodology and the facilitation guides.

Data collection took place during May 16–19, 2016. On the first two days, six data collectors visited their assigned CHCs and, on the third day, visited a convenience sample of health posts associated with the assigned CHCs. The remaining data collectors were assigned to the HNGV and private clinics. The two qualitative data collectors covered all the health facilities included in the sample.

All tablets and survey forms were password-protected. Only the assessment coordinator had access to all the surveys. Quantitative data were routinely uploaded and backed up to a secure network. Paper notes were destroyed once backed up electronically. Qualitative data notes were recorded on paper and later typed up for analysis.

### 2.5. Study population

The primary unit of analysis was children <24 months. Care-givers who were accompanied by a child who appeared to be <24 months of age were approached as they were exiting health facilities and requested to participate in a survey. If a caregiver was accompanied by more than one eligible child, questions were asked about the youngest child. Researchers abstracted only vaccination dates recorded in the official or temporary mother and child home-based record booklet/*Livrinho Saúde Inan ho Oan* (LISIO) or in the health center’s vaccination registers. No verbal reports of vaccination status or vaccination dates were accepted. All health center staff members were invited to participate in a self-administered KAP survey, independent of the department in which they routinely work. Although all health professionals (nurses, midwives and doctors) are competent to provide immunization services, the majority of EPI focal points in Timor Leste health facilities are nurses or midwives. Each team of data collectors aimed to complete a total of 50 exit interviews and 25 health worker KAP surveys over the four days of data collection. Caregiver exit interviews lasted approximately 20 min, while KAP surveys took 15 to 30 min to complete. All quantitative data were collected in Tetun.

For the FGDs, caregivers and health workers were selected from the same health facilities identified for quantitative data collection (excluding health posts). To reduce bias, FGDs were conducted on a different day, and participants in the quantitative arm were excluded from the qualitative interviews. Caregivers who were accompanied by a child who appeared to be <24 months of age were requested to participate as they were exiting the health center; age was then verified as reported by the caregiver. Caregivers were approached until the target of 4–6 participants per FGD session was reached. All health workers working at the selected health center on the day of qualitative data collection were also invited to participate in the health worker FGD, regardless of their involvement in vaccination services. Key informants for IDIs were identified among health administrators at selected health centers. FGDs were moderated and IDIs conducted in English using an English-Tetun interpreter. The qualitative team aimed to conduct one caregiver FGD, one health worker FGD, and two key informant IDIs at each health facility.

### 2.6. Data analysis

All quantitative data were extracted in Excel format from the electronic data collection platform and analyzed using Stata (version 14.2, College Station, Texas). The researchers created an eligibility tree to determine the total number of children with documented vaccination dates, those due at least one vaccine dose, and those with at least one MOV [15]. Frequency distributions were created for each variable from the caregiver exit interviews (among children with documented vaccination dates or documented evidence of non-vaccination) and the health worker KAPs. The final number of children with documented vaccination dates was determined from two sources, either the LISIO or the health facility register. Analyses using vaccination dates excluded children with illegible or invalid dates (either recorded incorrectly in the LISIO or facility register or on the questionnaire by the data collector).

Researchers then assessed MOV based on the child’s age on the date of interview, eligibility for vaccines in the national schedule, and contraindications (as reported by the caregiver). MOV were calculated among children who were eligible for at least one vaccine dose without valid contraindications, as per the national policy. The MOV estimate is a measurement of the inefficiencies of the health service in immunizing all eligible children [15]. Next, MOV was stratified by reason for visit and by vaccine type. All vaccines in the national schedule, with the exception of hepatitis B birth dose, inactivated polio vaccine (IPV), second dose of measles and rubella (MR2) vaccine, and one booster dose of DTP vaccine, were included in the calculation of MOV. These vaccines were excluded because they had only recently been introduced in Timor Leste and were not yet widely available at the time of data collection.

Researchers used the documented birth and vaccination dates to assess timeliness of vaccination. Extrapolating from past timeliness studies and using the nationally recommended ages for vaccination in Timor Leste, intervals for early, timely, and late vaccination were created for each of the vaccines in the national schedule, with the exception of recently introduced vaccines (hepatitis B birth dose, IPV, MR2, and one booster dose of DTP vaccine). Grace periods for the timely and late categories were included (Table 1) [22,23]. Intervals for administering all antigens, with the exception of bacille Calmette-Guerin (BCG) vaccine and oral polio vaccine (OPV) birth dose, do not have upper age limits in line with the national policy. BCG and OPV birth dose do have upper age limits per the national policy to not administer these vaccines after 365 days.

Researchers conducted a thematic analysis of the qualitative data and the comments fields of the quantitative surveys. The qualitative study team extracted key themes through an iterative process of re-readings of the notes from the FGDs, IDIs, and comments fields. Final themes were determined by consensus of the MOV assessment team.

### 2.7. Ethical approval

The Timor Leste Ministry of Health (MoH) Human Research Ethics Committee, reviewed all the study documents and considered the study protocol to be exempt as it was a program assessment. The study team included a verbal consent procedure before administering surveys or conducting FGDs or IDIs to ensure that participants had the opportunity to understand the assessment procedure and to decline participation. They were informed that participation was voluntary, they could leave the assessment at any time, and could choose to not answer any questions without repercussion. As there was no personally identifiable information collected, the Human Research Ethics Committee considered verbal consent to be appropriate as participation posed minimal risk to the participants. All participants in FGDs and IDIs also gave verbal consent for their direct quotes to be used in a manuscript.

## 3. Results

**Fig. 1.**
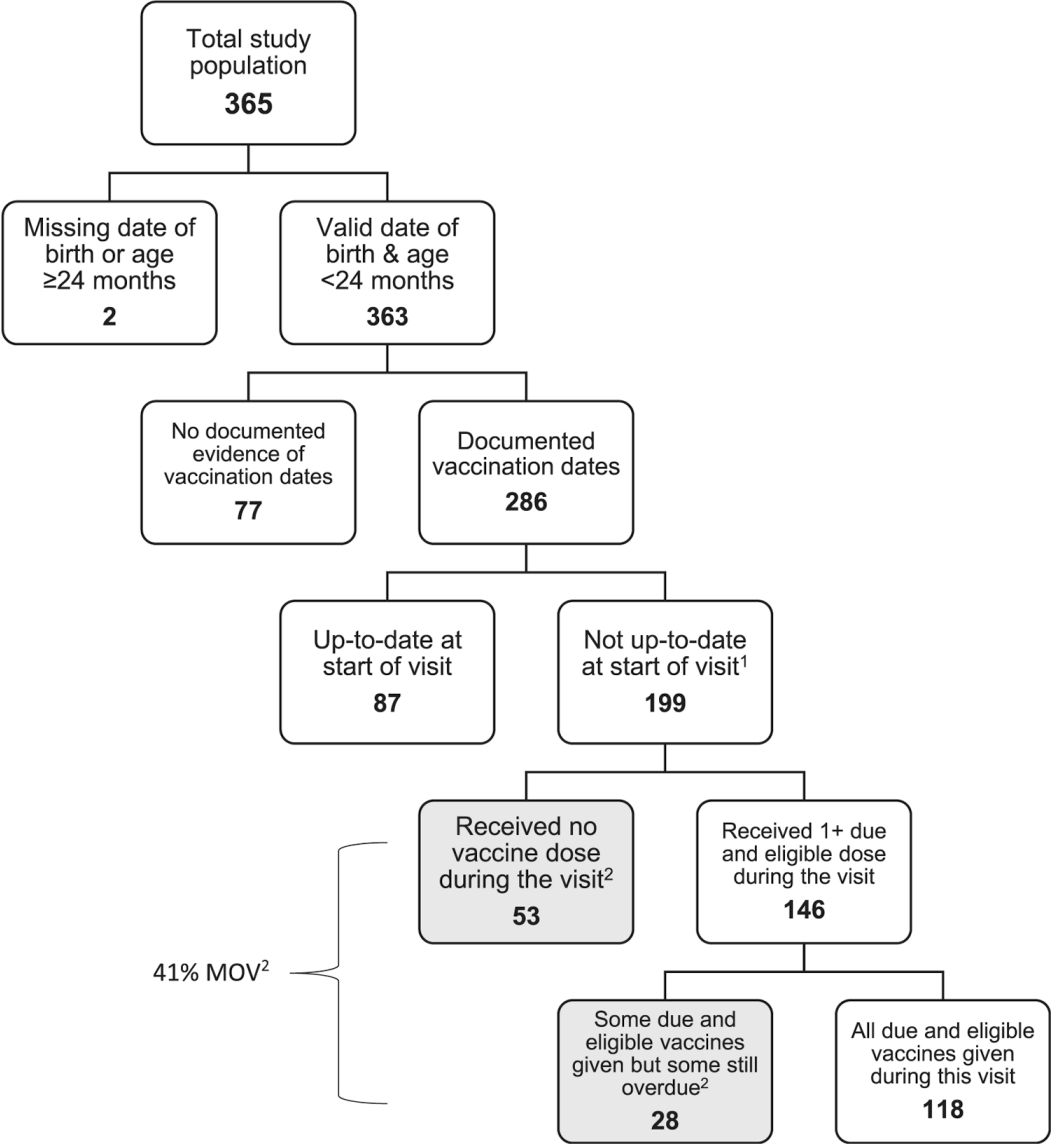
Health-center-based flow-chart for determining missed opportunities for vaccination (MOV), Timor Leste, 2016. ^1^All children were without contraindications. ^2^Missed opportunity for vaccination (MOV): contact with health services by a child (or adult) who is eligible for vaccination (unvaccinated, partially vaccinated/not up-to-date and free of contraindications to vaccination), which does not result in the individual receiving all vaccine doses for which he/she is eligible [13–15]

During four days of data collection, 11 data collectors completed 365 caregiver exit interviews (Fig. 1, Table 2) and 169 health worker KAPs at eight health centers. Data collectors completed a median of 50 caregiver surveys per health center and its associated health post (range: 10–67) and a median of 21 health worker KAPs per health center (range: 9–38) (data not shown in tables). There were no refusals to participate in the survey.

Due to logistical difficulties, the qualitative team only conducted four FGDs with caregivers, two FGDs with health workers, and two IDIs with health center administrators.

### 3.1. Caregiver interviews and FGDs

#### 3.1.1. Demographics

Of the 365 caregiver interviews conducted, 286 (78%) of the children had a documented vaccination date either recorded in the official or temporary LISIO or in the health center’s vaccination registers (Fig. 1); <5 were excluded because of illegible or invalid documented dates (data not shown). Approximately three-quarters (n = 218) of the interviews among caregivers with children aged <24 months and with documented vaccination dates were conducted in the five public CHCs and the HNGV, and the remaining (n = 68) were conducted in the two private facilities (Table 2). The majority of caregivers interviewed were mothers (93%), and most had some education (92%).

#### 3.1.2. Vaccination and caregiver attitudes

More than half of the children with documented vaccination dates were at the health center for a vaccination visit (164/286; 57%), and 87% had their LISIO in their possession at the visit (Tables 2 and 3). Among caregivers who did not have their LISIO, one indicated that they “never bring [LISIO] if not visiting for vaccination and growth monitoring.” (Exit interview, comments field) Among those whose children were vaccinated during the visit, most (92%) stated that they were informed of their child’s next vaccination appointment date. A caregiver during a FGD said, “The health workers always explain when the next doses are due.” How ever, only one-quarter (25%) of caregivers were told about potential adverse events following immunization (AEFI). In general, caregivers had positive attitudes toward vaccination and believed that vaccination was beneficial: “Vaccines are always available. We need to take our children,” said one caregiver during a FGD, and another stated, “Vaccination is good… not cause any disease.”

When caregivers were asked how the health center could improve, 29% cited the desire for better information on the vaccines administered, the diseases that the vaccines protected against, and AEFI: “… mothers don’t understand about the immunization.” (Exit interview, comments field) Additionally, mothers discussed how they feared AEFI, particularly because they felt as though some health workers were not well-trained enough to respond to adverse events, and “husbands complain of fever and AEFI.” (Care-giver FGD) Caregivers also cited the need for expanded and more flexible vaccination hours and days (14%), with some mothers asking for more home visits and regular outreaches: “…ensure nurses/midwives go to the SISCa to find and track defaulters and announce outreach days ahead, so mothers are ready.” (Caregiver FGD) One-quarter of the exit interview respondents suggested making more personnel available (27%) and reducing wait times (26%) as a way to improve services (Table 2), with 9% of respondents indicating both (data not shown). This sentiment was also echoed in caregiver FGDs: “Reduce the long wait!” (Caregiver FGD) The qualitative data showed some complaints about the waiting room facilities: “The waiting room for vaccination is not so good, waiting under sunshine and just call through window.” (Exit interview, comments field) Caregivers would like an improvement in health worker attitudes, as some caregivers are discouraged by their demeanor: “If mothers give birth at home, health workers get mad and refuse to vaccinate, which leads to delays.” (Caregiver FGD) Caregivers also cited some preferential treatment: “We are not satisfied. [Health workers] are unfair to those without a relative in the CHC.” (Caregiver FGD)

#### 3.1.3. Missed opportunities for vaccination (MOV)

Among all children with documented vaccination dates, 70% (199/286) were eligible for one or more vaccine doses during their health center visit (Fig. 1; Table 3). Some caregivers reported perceived contraindications which included a cough and/or cold, diarrhea, and malnutrition or anemia (data not shown), but no children were excluded from the analysis as a result of a reported valid contraindication (Fig. 1). Eighty-two percent (235/286) of children with documented vaccination dates had their dates recorded from their LISIO (data not shown). The remaining 18% (51/286) were documented either from the health facility register (36/286) or the source was not recorded in the questionnaire (15/286) (data not shown). During the visit, 118 of the 199 eligible children were vaccinated with all eligible doses, leaving 81 eligible children unvaccinated or under-vaccinated because of MOV. Therefore, among the children who were not up-to-date prior to the visit and were eligible for at least one vaccine dose, 41% (81/199) had a MOV (Fig. 1, Table 3). Among those at the health center for a vaccination visit, 32% (48/151) had a MOV, compared with 69% (33/48) of those attending the health service for a non-vaccination visit (Table 3).

Among children eligible for vaccination at their visit, 353 doses were due, of which 31% were missed (108/353) (Table 4). The largest percentage of missed doses were for OPV birth dose and measles and rubella vaccine first dose (MR1), with 65% (31/48) and 51% (21/41) of doses missed, respectively.

#### 3.1.4. Timeliness

Timeliness of vaccine doses among children with documented vaccination history varied by vaccine and dose, with timely administration of vaccines ranging from 64% to 90% (Table 4). Vaccine doses that were recommended later in the series were given in a less timely manner; for instance, timeliness of pentavalent vaccine fell from 77% for the first dose to 65% for the third dose. Nonetheless, MR1, given optimally at nine months, was given in a timely manner 90% of the time (36/40).

### 3.2. Health worker interviews, FGDs, and IDIs

#### 3.2.1. Demographics

Health workers participating in the KAP surveys included clinicians, nurses, midwives, and nursing assistants; the majority were nurses or midwives (65%) (Table 5). Of participating health workers, 43% had four years or fewer of clinical experience, whereas 19% had more than 20 years of experience. About half (45%) had previously been trained in vaccination or vaccine-preventable diseases, and 56% stated that their health center had opportunities for clinical or academic training sessions as part of continuing education.

#### 3.2.2. Knowledge, attitudes, and practices

One-third (33%) of the health workers believed their knowledge of vaccination to be insufficient. This was echoed during the FGDs and IDIs, where health workers cited being particularly unfamiliar with protocols regarding delayed vaccinations. Health workers also expressed confusion about the national guidance on “over-aged” children (children beyond recommended ages for vaccination, but who have not yet received any previous vaccinations or have delayed doses): “We need training on immunization.” (Health worker KAP, comments field) Trainings should also expand beyond Expanded Programme on Immunization (EPI) staff: “The nurses in health posts also need training on vaccination,” “As doctors we need training on vaccination,” and “The government people get sufficient training; private clinic [staff] do not.” (Health worker KAP, comments field) Health workers also expressed the need for training when new vaccines are introduced: “If there is a new vaccine, need to brief all health staff, and the new vaccine must [be] announced through media.” (Health worker KAP, comments field)

Additionally, 40% of health workers said they feared AEFI (Table 5). When asked about valid contraindications to vaccination, only 24% were able to identify pneumonia and other serious diseases from a multiple choice, multi-select list of options (which also included local reaction to a previous dose, low-grade fever, and seizures under medical treatment).

About half (46%) of all participating health workers reported that completing vaccination registers delayed delivery of vaccines (Table 5). During FGDs, health workers also discussed the need for improving the current recording and tracking systems, particularly for follow-up across health facilities: “Encourage better reporting by the district head office to centralized data.” (Health worker FGD) There was also a call for inclusion of all health facilities in the reporting systems, especially private health facilities: “In Dili, private clinics do not report to the CHC/district [level].” (Health worker FGD)

Forty-two health workers’ regular duties included administering vaccines (Table 5). The majority of health workers whose regular duties include administering vaccines believed that the health center was adequately staffed for immunization (86%, 36/42) and had enough vaccine vials for all patients in need (93%, 39/42). However, qualitative data collection revealed that vaccines were not available at all the facilities that were sampled. For example, only newborn vaccines are available at the HNGV due to a policy requirement. Caregivers expressed their desire for the HNGV to offer all vaccination services on a regular basis: “If possible, have vaccines in hospital too.” (Exit interview, comments field) Because vaccines are not available, caregivers have to make other arrangements for their infants to receive vaccinations: “We always get vaccines in CHC, because there are no vaccines in hospital.” (Exit interview, comments field) Additionally, vaccines were not available at one health facility because of nonfunctional cold chain equipment: “No vaccination in this clinic! If we had the vaccines, we’d give it, even to accompanying children… we need adequate storage… fridge seal is broken.” (Key informant IDI) Furthermore, health workers were somewhat hesitant to open vials for only one child: “Because only one baby, they could not provide the BCG vaccination to the baby.” (Exit interview, comments field)

## 4. Discussion

Timor Leste was the first country in the Southeast Asia Region to implement the updated WHO MOV methodology and the third country globally, following Chad and Malawi [21]. In addition to being the youngest country in the region, Timor Leste was selected to carry out this MOV assessment because it was considered a lower-performing country in the Southeast Asia Region. Through this assessment, researchers found that 41% of eligible children had a MOV during the health services encounter. Through the collection of both quantitative and qualitative data, researchers were able to identify areas of focus for improving the health system, including measures for increasing caregiver knowledge; strategies for improving health worker KAPs related to eligibility, contraindications, and management of AEFI; and systems changes so that vaccine and vaccination-related supplies are available and functioning and immunization status checks are done routinely.

Strategic investments in both caregiver and health worker education are needed. Among caregivers, the researchers documented their lack of understanding of which vaccines were included in the national schedule. Caregivers repeatedly cited the desire for increased education in this area. Improving health promotion in health facilities and communities is critical for addressing care-givers’ poor understanding related to immunization issues. Limited knowledge was also echoed in the health worker KAPs and qualitative research. Health workers reported knowledge gaps on national immunization policies and guidelines, especially regarding delayed vaccination, whether or not and when to open multi-dose vials. They also reported being unfamiliar with newly introduced vaccines; in 2016, Timor Leste introduced hepatitis B birth dose, IPV, measles and rubella vaccine, and one booster dose of DTP vaccine. Both caregivers and health workers also expressed concerns related to AEFI. The MoH, in coordination with other local partners, should explore strategies for augmenting health worker KAPs on vaccine contraindications and policies for catch-up of delayed vaccinations. Trainings that include both EPI and non-EPI staff are needed to raise awareness about immunization among health workers; such knowledge is expected to have a positive spillover effect to caregivers [24].

The researchers also documented systems issues, such as the lack of a working refrigerator at one health center that serves a high proportion of the community; limited availability of vaccination services on weekends and holidays; and an overall setup and patient flow in several health facilities that negatively impacted cross-departmental referrals. Replacing an old refrigerator with a functioning refrigerator is a potential quick win in reducing MOV by ensuring adequate cold storage for the necessary vaccines. The HNGV offered vaccination services only to newborns. Once discharged from the hospital, caregivers had to go to their local clinic to vaccinate their children with the remaining antigens in the infant schedule. Given that a high proportion of patients from Dili and beyond regularly receive non-vaccination services at the HNGV, making the necessary national policy changes to enable vaccine delivery at the HNGV could significantly improve the immunization status of many children, some of whom are referred from distant districts. Additionally, because the high proportion of MOV in this study was driven by the lack of vaccination services at the two health facilities with the highest patient loads, other districts are expected to have lower MOV proportions. The MoH should continue to explore innovate solutions to ameliorate the impact of the policy bottle-neck of not providing vaccination services at the HNGV. Other potential strategies for reducing MOV and increasing coverage may include updating national policies; improve LISIO ownership and ensure vaccination checks at all visits, irrespective of the reason for the visit; reorganize health facilities to provide vaccines; and streamline the vaccination and referral processes in the bigger CHCs (such as establishing a pre-registration triage and vaccination check-and-refer system).

### 4.1. Action steps to reduce MOV following the field assessment

The WHO methodology recommends conducting a brainstorming session immediately following the field data collection. The brainstorming session in Timor Leste consisted of three working groups and brought together the data collectors for the MOV assessment, MoH staff members from both immunization and child health departments, and local and international partners, to develop an action plan with interventions to reduce MOV based on their findings from the field. Following the brainstorming session, the field team led a high-level national debriefing to endorse the action plan and ensure funding and sustainability of the proposed interventions.

Since 2016, Timor Leste has worked to implement many of the interventions outlined in its MOV action plan. A desk review conducted in early 2018 by WHO Timor Leste country office and WHO Headquarters staff on the status of implementation showed that 65% of the proposed interventions had so far been partly or fully implemented within two years of field work. The interventions followed a three pronged approach targeting caregivers, health workers, and health systems. Non-functional cold chain equipment was promptly replaced following the field assessment, and distribution of refrigerators was expedited to health centers that did not have them. Timor Leste started regular health promotion and formal social mobilization meetings. All CHC and hospital staff members participated in a series of trainings on immunization during 2016–2017 that included pre- and post-tests, role playing, and practical demonstrations. These health promotion activities were evaluated in late 2017 for uptake and impact. Dili Municipality has also instituted performance-based incentives where best-performing health posts and CHCs in Dili Municipality are rewarded annually.

Additionally, Timor Leste plans to conduct additional trainings to educate health workers on the importance of recording vaccine doses in the LISIO and the importance of facilitating LISIO ownership among caregivers. Timor Leste also institutionalized monthly supportive supervision visits using a newly developed checklist with items specific to MOV. To address health systems challenges, Timor Leste implemented a monthly card-based defaulter tracking system, relocated the vaccination areas in certain clinics to streamline health center flow, and expanded selected CHC hours to include weekend hours.

Timor Leste continues to demonstrate its commitment to immunization activities with support from Gavi. Since the assessment in 2016, Timor Leste has capitalized on several different types of Gavi support, including a graduation grant and Health Systems Strengthening funds, to support the implementation of these MOV interventions. Similarly, the MoH plans to scale up successful MOV interventions beyond Dili Municipality, especially in the five referral hospitals with high patient volume and minimal vaccination services.

### 4.2. Study limitations

As stated in the MOV methodology, due to the sampling methodology, this assessment was not intended to be nationally representative or representative of the Dili Municipality. As such, it should be considered as a program assessment to identify areas of improvement for reducing MOV. In addition, while the questionnaires had been piloted and adapted to the country-context, there were still areas in which they could have been improved; questions may have been indicated as single-response, where a multiple-response option would have been more appropriate; also, some response choices lacked clarity, and some responses warranted an “other” response option with the option to specify an answer. Similarly, although there were efforts to ensure that caregiver exit interviews and focus group discussions were conducted away from the earshot of health workers or higher-ups, these were conducted within the vicinity of health facilities due to logistical constraints. As a result, the responses we received may provide a more favorable view of the health facility and quality of services received. Finally, the estimation of MOV was limited to children with documented vaccination dates, either from their LISIO or the health center register. We would expect children without documentation to be more likely to have had a MOV, so the true estimate of MOV in Dili is likely to be higher [13].

## 5. Conclusion

In a young country like Timor Leste, ensuring that health remains a priority is important for building strong and sustainable human and economic capital. Timor Leste was the first country in the Southeast Asia Region to implement the updated WHO MOV methodology. The MOV assessment has shown that 41% of children eligible for one or more vaccines and who visited the health facilities on the day of the assessment had a preventable MOV. In Timor Leste, as in other countries, there are several low-hanging fruit opportunities to increase the efficiencies of the vaccination programs. Since the MOV assessment, Timor Leste has continued to make substantial efforts to strengthen its immunization program through implementation of activities to reduce MOV. These activities contributed to the country’s recent achievement of verifying the elimination of measles, rubella, and congenital rubella syndrome in 2018. Measures to reduce MOV and improve health system efficiencies must continue to be scaled up across the country. Furthermore, the use of the updated WHO methodology has shown that it is a low resource intensive strategy that is able to provide a wide-range of actionable solutions. Other countries in the region could learn from the experience of Timor Leste in conducting an assessment of MOV and implementing successful interventions. Given the flexibility of the updated methodology, it lends itself easily to adaptation to different country contexts.

## Figures and Tables

**Table 1:** Time intervals used for classifying timeliness of vaccine doses received by surveyed children, using the nationally recommended ages for vaccination, Timor Leste, 2016.^1^

Vaccine	Scheduled age of vaccination	Too early	Timely	Late
**Birth dose**				
BCG^2^	Birth	-	0–30 days	30–365 days
OPV^3^			0–14 days	14–365 days
**First dose**	6 weeks (42 days)	<42 days	42–56 days	≥57 days
OPV^3^				
Pentavalent vaccine^4^				
**Second dose**	10 weeks (70 days)	<70 days	70–84 days	≥85 days
OPV^3^				
Pentavalent vaccine^4^				
**Third dose**	14 weeks (98 days)	<98 days	98–112 days	≥113 days
OPV^3^				
Pentavalent vaccine^4^				
**MR1**^5^	9 months (270 days)	<270 days	270–365 days	≥366 days
				

1 The table does not comprehensively include all vaccines listed in the national immunization schedule for children <24 months; newly introduced vaccines (as of 2016) were excluded (hepatitis B birth dose, inactivated polio vaccine, measles and rubella vaccine second dose, and diphtheria-tetanus-pertussis vaccine fourth dose).

2 Bacille Calmette-Guerin (BCG) vaccine.

3 Oral poliovirus vaccine (OPV).

4 Diphtheria-tetanus-pertussis-hepatitis B-Haemophilus influenzae type b (pentavalent) vaccine.

5 Measles and rubella vaccine first dose (MR1).

**Table 2:** Characteristics of surveyed caregivers of children with documented vaccination dates, Timor Leste, 2016.

	n	%
	286	
***Child demographics***		
**Sex**	**275**	
Male	142	52
Female	133	48
**Age**	**286**	
<12 months	263	92
≥12 months	23	8
**Ever vaccinated**	**259**	
Yes	226	87
No	33	13
***Caregiver demographics***		
**Relationship to child**	**270**	
Mother	252	93
Father	17	6
Uncle/aunt/grandparent	1	<1
**Educational Level**	**278**	
None	23	8
At least some primary	45	16
At least some secondary	210	76
***Health center visit***		
**Type of Health Center**	**286**	
Public national hospital	35	12
Public community health center	183	64
Private or nongovernmental organization health center	68	24
**Child has home-based record**	**271**	
Yes, available at this visit	235	87
Yes, but not available at this visit	35	13
No	1	<1
**Did staff ask for the card?**	**255**	
Yes	187	73
No	68	27
***Knowledge and Attitudes***		
**How would you assess your level of knowledge on vaccines/vaccination?**	**277**	
Adequate	17	6
Fairly adequate	188	68
Inadequate	72	26
**Ever requested for but refused vaccination services?**	**273**	
Yes	57	21
No	216	79
**Told about vaccination reactions?**^1^	**169**	
Yes	127	75
No	42	25
**Informed of next vaccination date?**^1^	**169**	
Yes	155	92
No	14	8
**Satisfied with service today?**^1^	**169**	
Yes	164	97
No	5	3
**Suggestions for improving health center services**^2^	**171**	
Less of a wait	45	26
Hours and days when vaccinations are available should not be limited	24	14
Friendlier treatment of the public	19	11
Health centers should always have vaccination materials	19	11
More personnel should be available	46	27
Better information should be provided on vaccines given, diseases prevented, and reactions produced	49	29

1 Among caregivers who indicated that their child had been vaccinated on the day of the assessment.

2 Respondents allowed to select multiple responses.

**Table 3:** Prevalence of missed opportunities for vaccination (MOV)^1^ among surveyed children, by reason for visit, Timor Leste, 2016.

	On arrival for this health visit			During this health visit		After this health visit	
	Children with documented vaccination dates	Children with 1 + eligible doses due		Children vaccinated with all eligible doses during visit^2^		Children with 1 + MOV^2^	
	n	n	%	n	%	n	%
Vaccination visit	164	151	92	103	68	48	32
Non-vaccination visit	122	48	39	15	31	33	69
*Medical consultation*	49	22	45	5	23	17	77
*Healthy child visit or check-up*	41	11	27	2	18	9	82
*Child is accompanying adult*	3	3	100	0	0	3	100
*Hospitalization*	19	6	32	5	83	1	17
*Other*	6	5	83	2	40	3	60
*No reason reported*	4	1	25	1	100	0	0
**Total**	286	199	70	118	59	81	41

1 Missed opportunity for vaccination (MOV): contact with health services by a child (or adult) who is eligible for vaccination (unvaccinated, partially vaccinated/not up-to-date, and free of contraindications to vaccination), which does not result in the individual receiving all the vaccine doses for which he or she is eligible [13–15].

2 Among the subset of children with documented vaccination dates and eligible for one or more vaccine doses (n = 199).

**Table 4:** Timeliness of vaccine doses administered to surveyed children and missed opportunities by dose among surveyed children with documented vaccination histories, Timor Leste, 2016.

Vaccine dose	Timeliness^1^				MOV by dose		
	Total number of children who received dose^2^	Too early	Timely	Late	Eligible doses due	Eligible doses missed at visit	
		%	%	%	n	n	%
**Birth dose**							
BCG^3^	269	-	88	12	31	6	19
OPV^4^	237	-	89	11	48	31	65
**First dose**							
OPV^4^	216	7	79	14	41	9	22
Pentavalent^5^ vaccine	213	8	77	16	54	14	26
**Second dose**							
OPV^4^	163	7	71	22	29	3	10
Pentavalent^5^ vaccine	159	8	70	23	31	7	23
**Third dose**							
OPV^4^	121	4	64	32	35	7	20
Pentavalent^5^ vaccine	118	4	65	31	43	10	23
**MR1**^6^	40	5	90	5	41	21	51
**Total**					353	108	31

1 Please see Table 1 for intervals and immunization schedule used for this analysis.

2 Children with documented history of receiving a dose either on the day of survey or previously.

3 bacille Calmette-Guerin (BCG) vaccine.

4 Oral poliovirus vaccine (OPV).

5 Diphtheria-tetanus-pertussis-hepatitis B-Haemophilus influenzae type b (pentavalent) vaccine.

6 First dose of measles and rubella vaccine (MR1).

**Table 5:** Characteristics and knowledge, attitudes, and practices of surveyed health workers, Timor Leste, 2016.

	n	%
	169	
***Health worker demographics***		
**Sex**	**165**	
Male	34	21
Female	131	79
**Professional Training**	**162**	
Clinician	38	23
Nurse/Midwife	106	65
Nursing Assistant	9	6
Other	9	6
**Years of experience**	**169**	
0 to 4	72	43
5 to 9	40	24
10 to 14	16	9
15 to 19	9	5
20+	32	19
**Type of Service**	**169**	
Public	139	82
Private	30	18
**Ever trained in vaccination or vaccine-preventable diseases**	**169**	
Yes	76	45
No	93	55
**Opportunities for clinical or academic trainings as part of job**	**168**	
Yes	94	56
No	74	44
***Health worker knowledge, attitudes, practices***		
**My knowledge of vaccination and the EPI is sufficient to meet its needs**	**169**	
Agree	114	67
Disagree	55	33
**Contraindications for any vaccine**	**167**	
Local reaction to previous dose	7	4
Low-grade fever	40	24
Seizures under medical treatment	33	20
Pneumonia and other serious diseases	40	24
None of the above	47	28
**When should vaccination status be assessed?**	**164**	
Child’s wellness/routine visit	54	33
Consultation for any illness	37	23
When a child is accompanying an adult for any reason	29	18
All of the above	44	27
**Why is vaccination status incomplete for some children?**	**169**	
Parents’ negative beliefs related to vaccination	86	51
Hours of vaccination are incompatible with parents’ schedule	14	8
Health workers do not review children’s vaccination cards or vaccination status	9	5
False contraindications for vaccination by health workers	2	1
All of the above	58	34
**I fear adverse reactions to vaccines**	**169**	
Agree	68	40
Disagree	101	60
**Completing vaccination registers delays vaccination**	**169**	
Agree	77	46
Disagree	92	54
**What instructions do you give caregivers when you give them a new vaccination card?**^1,2^	**42**	
Keep the card safe	20	48
Bring this card to all visits to the health center	32	76
Bring this card only when you come for vaccinations	9	41
Other	1	4
**There is sufficient staff offering immunization services at this center**^2^	**42**	
Agree	36	86
Disagree	6	14
**There are enough vials of vaccine for all patients in need**^2^	**42**	
Agree	39	93
Disagree	3	7

1 Respondents were allowed to select multiple responses.

2 Only asked of health workers who administer vaccines as part of their job.
